# Risk factors and clinical presentation of coronary artery disease in young patients: a decade-long single-center experience

**DOI:** 10.1186/s12872-026-05729-5

**Published:** 2026-04-09

**Authors:** Marco Busco, Luigi Cappannoli, Francesco Bianchini, Cristina Aurigemma, Fabio Casamassima, Domenico Cicchella, Michele Marchetta, Donato Antonio Paglianiti, Francesco Fracassi, Antonino Buffon, Enrico Romagnoli, Mattia Lunardi, Rocco Antonio Montone, Antonio Maria Leone, Lazzaro Paraggio, Carlo Trani, Francesco Burzotta

**Affiliations:** 1 Department of Cardiovascular and Pulmonary Sciences, Università Cattolica del Sacro Cuore, Rome, Italy; 2 Department of Cardiovascular Sciences, Fondazione Policlinico Universitario A. Gemelli IRCCS, Rome, Italy; 3 Center of Excellence in Cardiovascular Sciences, Ospedale Isola Tiberina, Rome, Italy

**Keywords:** Coronary artery disease, Young patients, Invasive coronary angiography, Single center

## Abstract

**Background:**

Coronary artery disease (CAD) is a major health concern that also affects younger individuals, yet data on this population remain limited. We aimed to analyze trends, clinical features, and risk factors in patients aged ≤ 40 years undergoing invasive coronary angiography (ICA) at a high-volume Italian center.

**Methods:**

We retrospectively analyzed all patients aged ≤ 40 years who underwent ICA from January 2010 to March 2024, assessing demographics, cardiovascular risk factors (CVRFs), and clinical presentations. We compared patients with significant CAD requiring revascularization and those without, exploring gender and ethnic differences.

**Results:**

Of 38,304 ICA procedures, 441 (1.2%) involved young patients, with a decreasing trend over time. Males comprised 74.1% of the cohort. CAD requiring intervention was found in 100 procedures (22.7%), while 341 (77.3%) had no significant disease. The most prevalent CVRFs were hypertension (30.6%), active smoking (28.8%), family history of ischemic heart disease (26.8%), and hypercholesterolemia (24.9%). Diabetes prevalence was lower (8.6%). CAD patients had a higher CVRF burden, except for diabetes, which showed no significant difference. Notably, 10.0% of CAD patients lacked traditional CVRFs, while peripheral artery disease was more prevalent in patients with CAD. In particular, non-Caucasian patients had higher rates of hypertension, hypercholesterolemia, diabetes, and CAD. Males had higher rates of ST-segment elevation acute coronary syndrome and CAD, whereas nearly half of females had no CVRFs. Results were consistent in a sensitivity analysis restricted to the first procedure per patient.

**Conclusions:**

In very young patients undergoing ICA, age and hypercholesterolemia were the only independent predictors of angiographic CAD, while ethnicity was not independently associated after multivariable adjustment. Clinical presentation—particularly acute coronary syndromes and typical angina— were strongly associated with obstructive disease.

**Graphical Abstract:**

**Figure:**
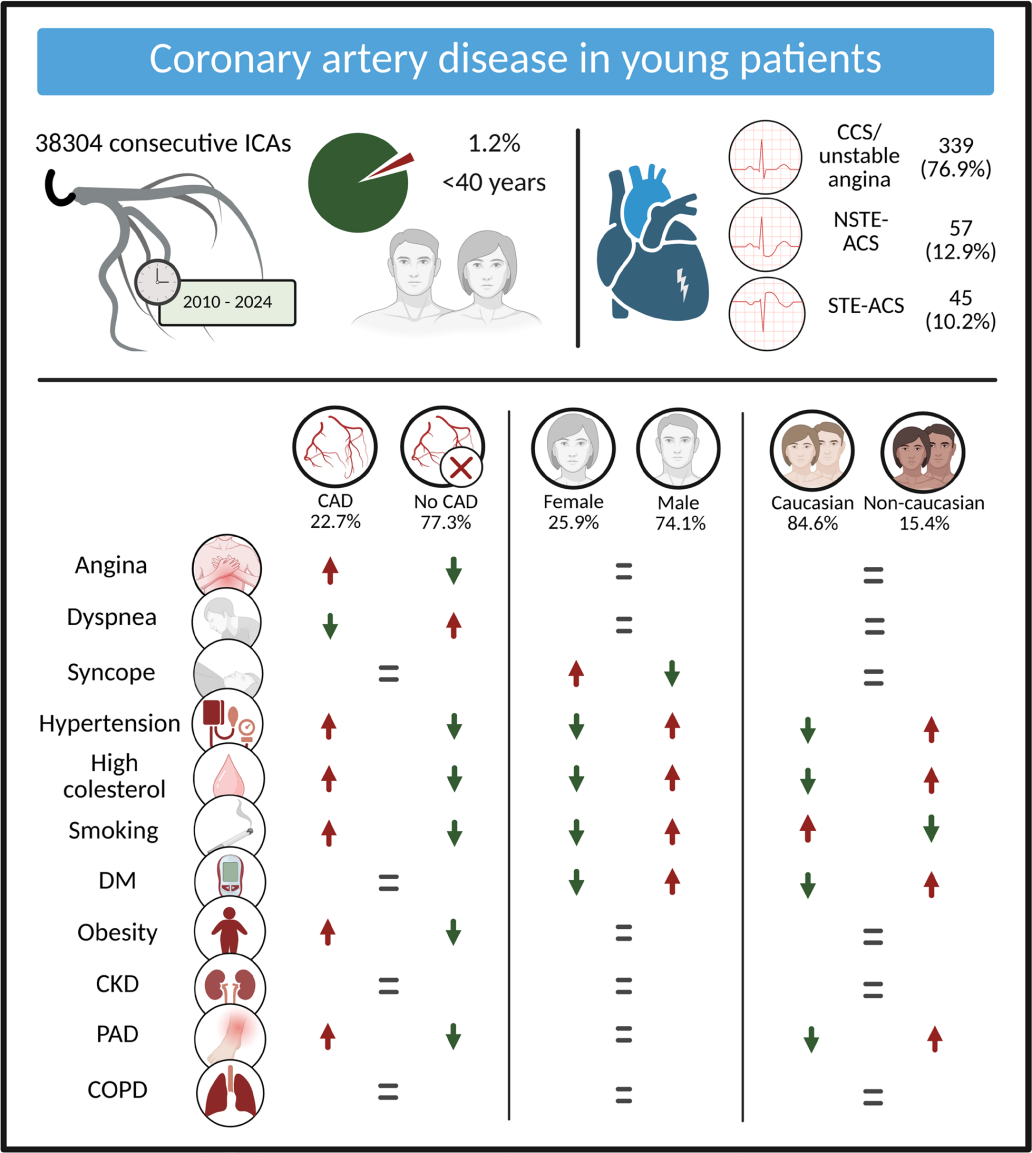
Coronary artery disease in young patients. Prevalence of major CVRFs in patients with and without CAD, stratified by sex and ethnicity

**Supplementary Information:**

The online version contains supplementary material available at 10.1186/s12872-026-05729-5.

## Introduction

Coronary artery disease (CAD) is a significant health concern even among younger individuals, though its low prevalence in this population has resulted in a paucity of data. Despite this, CAD in a younger population is associated with considerable morbidity, psychological impact, socioeconomic burden, and a notable loss of Disability-Adjusted Life Years (DALYs), as it affects people in their most productive years [1, 2].

The age limit for defining a “young population” varies in the literature; some studies establish a threshold of 45 years [3, 4], while others designate it at 40 years [5, 6]. For our study, we have opted for a threshold of 40 years to select a very young population and to have a better definition of clinical characteristics and risk factors with a heavy impact from the earliest age.

While age is widely recognized as a significant determinant for cardiovascular disease (CVD), traditional risk factors, such as active smoking, hypercholesterolemia, hypertension, diabetes, and family history of CAD, have a more pronounced impact on younger individuals [7].

Approximately 30 years of age appears to represent a threshold for these risk factors to start having a major impact on the population. In fact, in 15-19-year-olds, only 2% of males and 0% of females exhibited major risk factors, in contrast to the 30–34 age group, where they escalated to 20% and 8%, respectively [8]. The distribution and predictive power of cardiovascular risk factors (CVRFs) for acute myocardial infarction (MI) differs across studies. The INTERHEART study [7], showed that the classic CVRFs had a greater relative impact on the risk of acute MI in younger individuals compared to their older counterparts. This finding suggests that many premature MIs could be preventable, highlighting the potential for prevention in younger populations.

The Framingham study [9] reported a 10-year MI rate of 12.9 per 1000 men aged 30–34 and 5.2 per 1000 women aged 35–44. Globally, it is estimated that 2–6% of patients with MI are younger than 40 years old [10, 11]. Although alternative diagnoses often receive more attention, CAD accounts for over half of all sudden cardiac deaths (SCDs) in individuals in their fourth decade of life [12, 13]. Furthermore, the incidence of acute MI in younger patients has significantly increased over the past two to three decades, as demonstrated by the Atherosclerosis Risk in Communities (ARIC) Surveillance, particularly in developing countries [14].

The most common clinical presentation of CAD in younger patients is ST-segment elevation acute coronary syndrome (ST-ACS), along with single-vessel disease as identified through invasive coronary angiography (ICA) [5, 15].

The aim of the present study is to characterize the temporal trend, the clinical features and the risk factors of individuals aged 40 years or younger who underwent coronary angiography at our institution’s catheterization laboratory (Cath-lab).

## Methods

### Study population

**Fig. 1:**
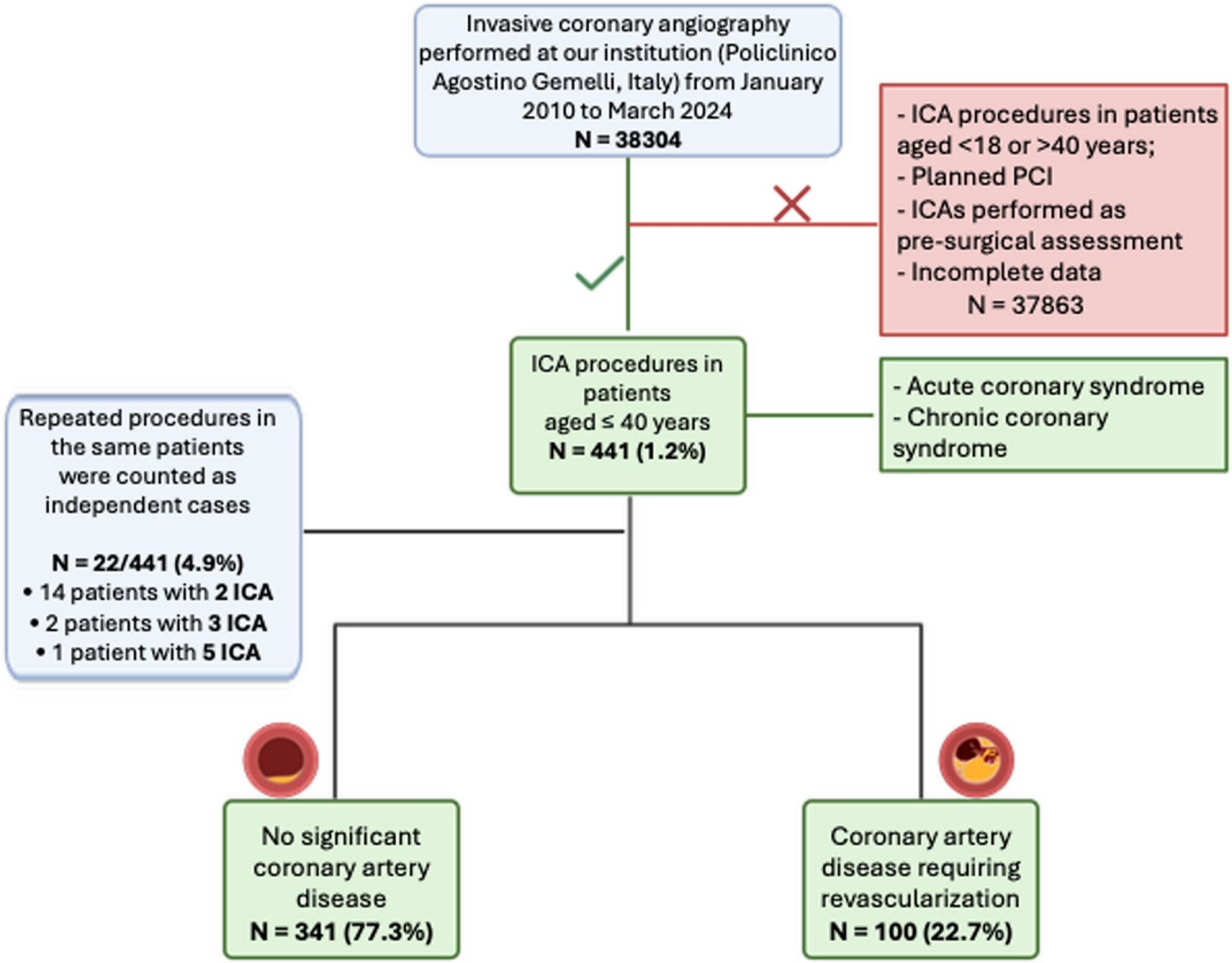
Flow-chart of patients’ selection

We conducted a retrospective analysis involving consecutive patients aged 40 years or younger who underwent ICA (in both the acute and chronic setting) at our institution (Policlinico Universitario Agostino Gemelli IRCCS, Rome, Italy) from January 2010 to March 2024 (Fig. 1).

Indications for ICA included suspected acute or chronic coronary syndrome. All patients underwent ICA according to the contemporary guideline recommendations valid at the time of the procedure and the treating cardiologist’s clinical judgment; when available and appropriate, non-invasive testing (exercise ECG, perfusion imaging, or coronary CT angiography) or functional tests were performed prior to ICA in accordance with prevailing local practice and guidelines.

The exclusion criteria were as follows: (1) procedures performed in patients aged < 18 years or > 40 years; 2) planned percutaneous coronary interventions (PCIs) performed without prior diagnostic angiography; (3) coronary angiographies undertaken solely for preoperative assessment; and (4) procedures with incomplete clinical or angiographic data.

Temporal trends in ICA procedures were first analyzed. Baseline clinical and demographic characteristics were collected, together with data on traditional cardiovascular risk factors and selected non-traditional risk factors (e.g., chronic kidney disease, active malignancy, coagulation disorders). Hypertension, diabetes mellitus, and hypercholesterolemia were defined based on at least one of the following criteria: (i) a documented prior physician diagnosis reported in the medical records, (ii) ongoing use of disease-specific medications, or (iii) diagnosis established at the time of admission for invasive coronary angiography according to contemporary clinical standards. Smoking status was defined according to patient-reported current or past cigarette smoking, and these categories were analyzed separately in descriptive analyses and included as covariates in regression models. Family history of ischemic heart disease was defined according to standard criteria as premature ischemic heart disease in a first-degree relative (age < 55 years in men and < 65 years in women), as documented in the medical records. The absence of traditional cardiovascular risk factors was therefore based on clinically documented diagnoses rather than on single admission measurements alone. Recreational drug use (cocaine and cannabis) was recorded based on clinical documentation and toxicology screening performed as part of routine clinical assessment in young patients with suspected acute coronary syndromes.

For the purpose of clarity and consistent terminology, patients were classified into two mutually exclusive groups for the main analyses: (1) Patients with significant coronary artery disease (CAD) defined as the presence of angiographically significant stenoses (≥ 70% diameter stenosis in a major epicardial vessel or ≥ 50% in the left main) or angiographically intermediate stenoses (40–69%) with documented objective evidence of myocardial ischemia (functional testing or invasive physiological assessment); and (2) patients without significant coronary artery disease (no-CAD), including individuals with completely normal coronary arteries, those with angiographic evidence of only mild atherosclerosis (lesions < 40%), or those with intermediate stenoses (40–69%) without objective evidence of ischemia. Non-atherosclerotic and alternative etiologies were identified when documented in the medical records, including spontaneous coronary artery dissection (SCAD), coronary vasospasm/Prinzmetal angina, microvascular angina, congenital coronary anomalies (e.g., anomalous coronary origin/course), myocardial bridging, and coronary fistula. These procedures were classified within the no-CAD group, as the primary endpoint was obstructive CAD based on angiographic stenosis criteria and/or objective evidence of ischemia.

The primary unit of analysis was the coronary angiography procedure. In patients who underwent more than one ICA before the age of 40 years, each procedure was initially considered an independent case, in order to reflect real-world clinical practice and the overall burden of invasive coronary investigations in young patients. Among the 441 procedures included in the analysis, 22 represented repeated angiographies performed in the same patients. Specifically, 14 patients underwent two procedures, 2 patients underwent three procedures, and 1 patient underwent five procedures during the study period. Consequently, the overall study cohort consisted of 419 unique patients and 441 procedures. To assess the potential impact of repeated procedures on prevalence estimates and associations, a sensitivity analysis including only the first coronary angiography per patient was subsequently performed.

The main regression analyses were performed at the procedure level and therefore assumed independence of observations; potential residual within-patient correlation due to repeated procedures cannot be entirely excluded.

Differences between procedures with significant coronary artery disease (CAD) requiring percutaneous coronary intervention or coronary artery bypass graft surgery and those without significant coronary disease were subsequently assessed.

To evaluate the potential role of ethnicity, patients were stratified according to Caucasian versus non-Caucasian origin. Ethnicity was recorded according to the information available in the medical records, based on administrative or self-reported data at the time of hospital admission.

This retrospective study was conducted in accordance with the Declaration of Helsinki. Ethical review and approval were waived by the Institutional Review Board of Fondazione Policlinico Universitario A. Gemelli IRCCS (Rome, Italy), in accordance with institutional policies for retrospective studies based on anonymized data. All patients had previously signed a general informed consent at the time of the procedure, allowing the use of anonymized clinical data for research purposes.

### Statistical analysis

Data are summarized as means (± standard deviation) or medians (with interquartile range) for continuous variables and as numbers and percentages for categorical variables. Chi-square (χ²) or Fisher’s exact test were employed to assess differences among categorical variables. Student’s t-test or Mann-Whitney U test was used to compare continuous variables, depending on the presence of normal distribution. The normality of continuous variables was formally assessed using both the Kolmogorov–Smirnov and Shapiro–Wilk tests; non-normally distributed variables were summarized as medians with interquartile range and analyzed using non-parametric tests.

Multivariable logistic regression analyses were performed to identify independent predictors of coronary artery disease. Separate models were constructed to evaluate (i) traditional cardiovascular risk factors and ethnicity, and (ii) clinical presentation and echocardiographic variables, adjusting for age and sex as a priori confounders. Given the relatively low number of events, the number of covariates included in each model was limited to preserve events-per-variable and reduce overfitting. Results are reported as adjusted odds ratios with 95% confidence intervals. A two-tailed p-value < 0.05 was considered statistically significant for all analyses. Statistical analysis was performed using IBM SPSS Statistics 30.

## Results

### Overview of patients

**Fig. 2:**
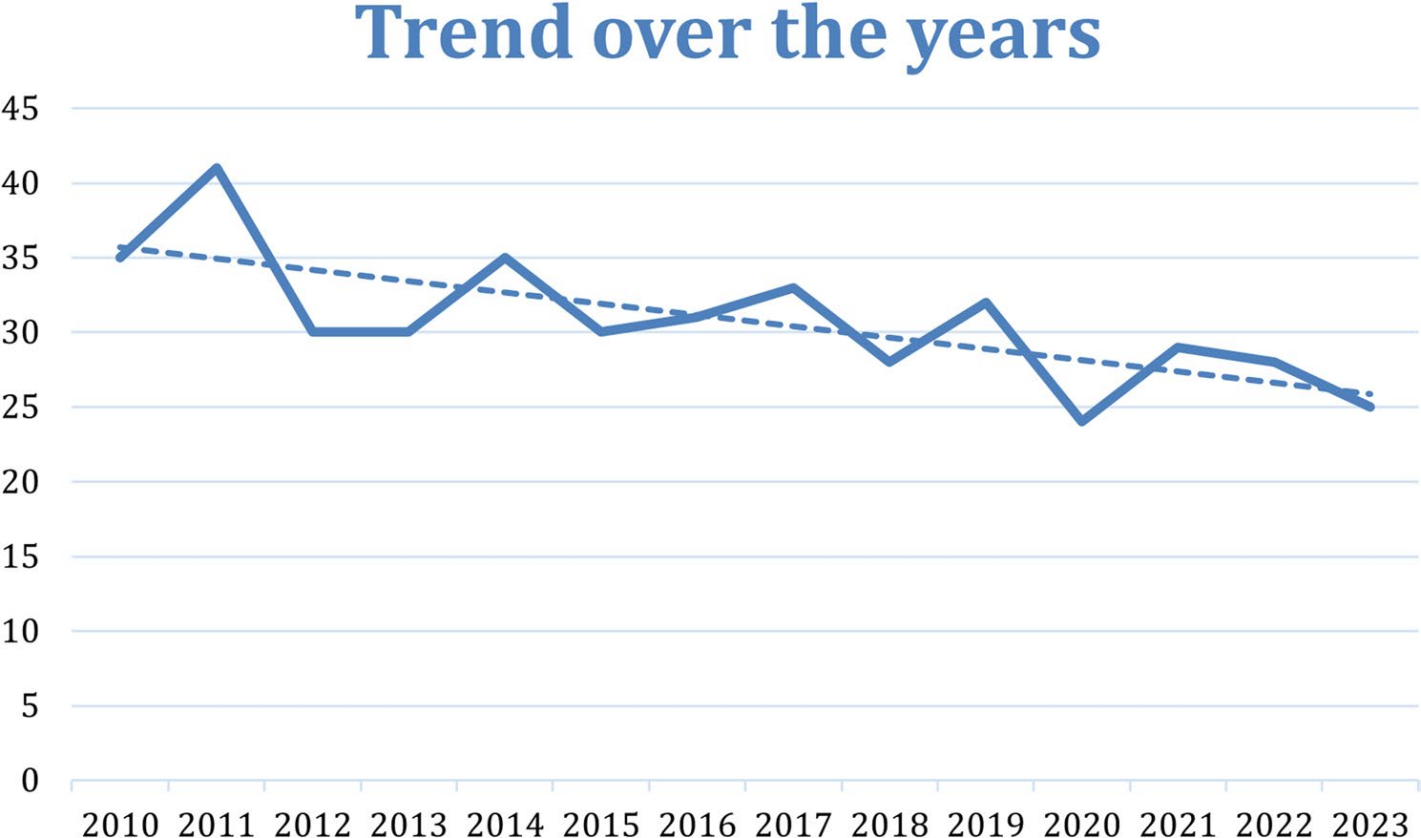
Trend of procedures over the years

Among 38,304 ICA procedures from 2010 to March 2024, 441 (1.2%) were performed in patients aged ≤ 40 years (including 22 repeated cases). The cohort was predominantly male (74.1%). Of all procedures, 100 (22.7%) showed CAD treated by PCI or CABG (CAD group), whilst 341 (77.3%) showed no significant coronary atherosclerosis (no-CAD group). Among no-CAD procedures, documented alternative etiologies included SCAD (*n* = 4), coronary vasospasm/Prinzmetal angina (*n* = 8), microvascular angina (*n* = 1), congenital coronary anomalies (*n* = 6), myocardial bridging (*n* = 4), and coronary fistula (*n* = 1); myocarditis (*n* = 13) and cardiomyopathies (*n* = 2) were also recorded as alternative diagnoses. Overall, a decreasing trend in the number of procedures over time was observed (Fig. 2). The mean age was 34 ± 6 years (37 ± 4 in the CAD group vs. 33 ± 7 in the no-CAD group, respectively). A total of 373 (84.6%) participants were Caucasian, while 68 (15.4%) belonged to other ethnic groups. Over the study period, a progressive increase in the number of non-Caucasian patients undergoing invasive coronary angiography was observed, whereas the number of Caucasian patients remained relatively stable (Supplementary Fig. 1). Baseline and demographic characteristics of the included patients are summarized in Table 1 and Table 2.

**Table 1:** Baseline characteristics

Baseline characteristics	All procedures (441)
Mean age, years	34 ± 6
No-CAD	77.3%
CAD	22.7%
Males	74.1%
Females	25.9%
Caucasian	84.6%
Other ethnic groups	15.4%

*Abbreviations: CAD* Coronary artery disease

**Table 2:** Distribution of cardiovascular risk factors in the study population according to the presence of coronary artery disease

Risk factors	All procedures (441)	No coronary disease (341; 77.3%)	Coronary disease (100; 22.7%)	*p*-value
Absence of risk factors	137 (31.0%)	127 (37.5%)	10 (10.0%)	**< 0.001**
Active smoke habit	127 (28.8%)	84 (24.6%)	43 (43.0%)	**< 0.001**
Past smoke habit	29 (6.6%)	24 (7.0%)	5 (5.0%)	0.470
Hypertension	135 (30.6%)	91 (26.7%)	44 (44.0%)	**0.001**
Hypercholesterolemia	110 (24.9%)	64 (18.8%)	46 (46.0%)	**< 0.001**
Familiarity for IHD	118 (26.8%)	78 (22.9%)	40 (40.0%)	**0.001**
Diabetes on oral therapy	14 (3.2%)	11 (3.2%)	3 (3.0%)	0.910
Diabetes on insulin therapy	24 (5.4%)	16 (4.7%)	8 (8.0%)	0.200
Peripheral artery disease	26 (6.0%)	15 (4.5%)	11 (11.1%)	**0.016**
Chronic kidney disease (eGFR < 60 ml/min/1.73 m^2^)	30 (6.7%)	23 (6.6%)	7 (7.1%)	0.864
Chronic obstructive pulmonary disease	10 (2.3%)	8 (2.4%)	2 (2.0%)	0.818
Obesity	38 (8.6%)	24 (7.0%)	14 (14.0%)	**0.029**
Coagulation disorder	21 (4.8%)	15 (4.5%)	6 (6.0%)	0.529
Chronic inflammatory disease	60 (13.6%)	44 (12.9%)	16 (16.0%)	0.430
Active cancer	9 (2.1%)	8 (2.4%)	1 (1.0%)	0.394
History of cancer	18 (4.1%)	14 (4.1%)	4 (4.0%)	0.937
Use of recreational drug	23 (5.2%)	19 (5.6%)	4 (4.0%)	0.534
Anemia	24 (5.4%)	21 (6.2%)	3 (3.0%)	0.243
Dysthyroidism	30 (6.8%)	26 (7.6%)	4 (4.0%)	0.221
Liver dysfunction	12 (2.7%)	10 (2.9%)	2 (2.0%)	0.504
Congenital heart disease	61 (13.8%)	57 (16.7%)	4 (4.0%)	**0.001**
STE-ACS	45 (10.2%)	6 (1.8%)	39 (39.0%)	**< 0.001**
NSTE-ACS	57 (12.9%)	30 (8.8%)	27 (27.0%)	**< 0.001**
Preserved EF (≥ 50%)	286 (64.9%)	252 (73.9%)	34 (34.0%)	**< 0.001**
Mildly reduced EF (30%≤EF < 50%)	58 (13.2%)	48 (14.2%)	10 (10.1%)	0.297
Severely reduced EF (EF < 30%)	29 (6.6%)	28 (8.2%)	1 (1.0%)	**0.010**

*Abbreviations: IHD* Ischemic Heart Disease, *eGFR* Estimated Glomerular Filtration Rate, *STE-ACS* ST-Elevation Acute Coronary Syndrome, *NSTE-ACS* Non-ST-Elevation Acute Coronary Syndrome, *EF* Ejection Fraction

Bold values indicate statistical significance (*p* < 0.05)

### Risk factors

**Fig. 3:**
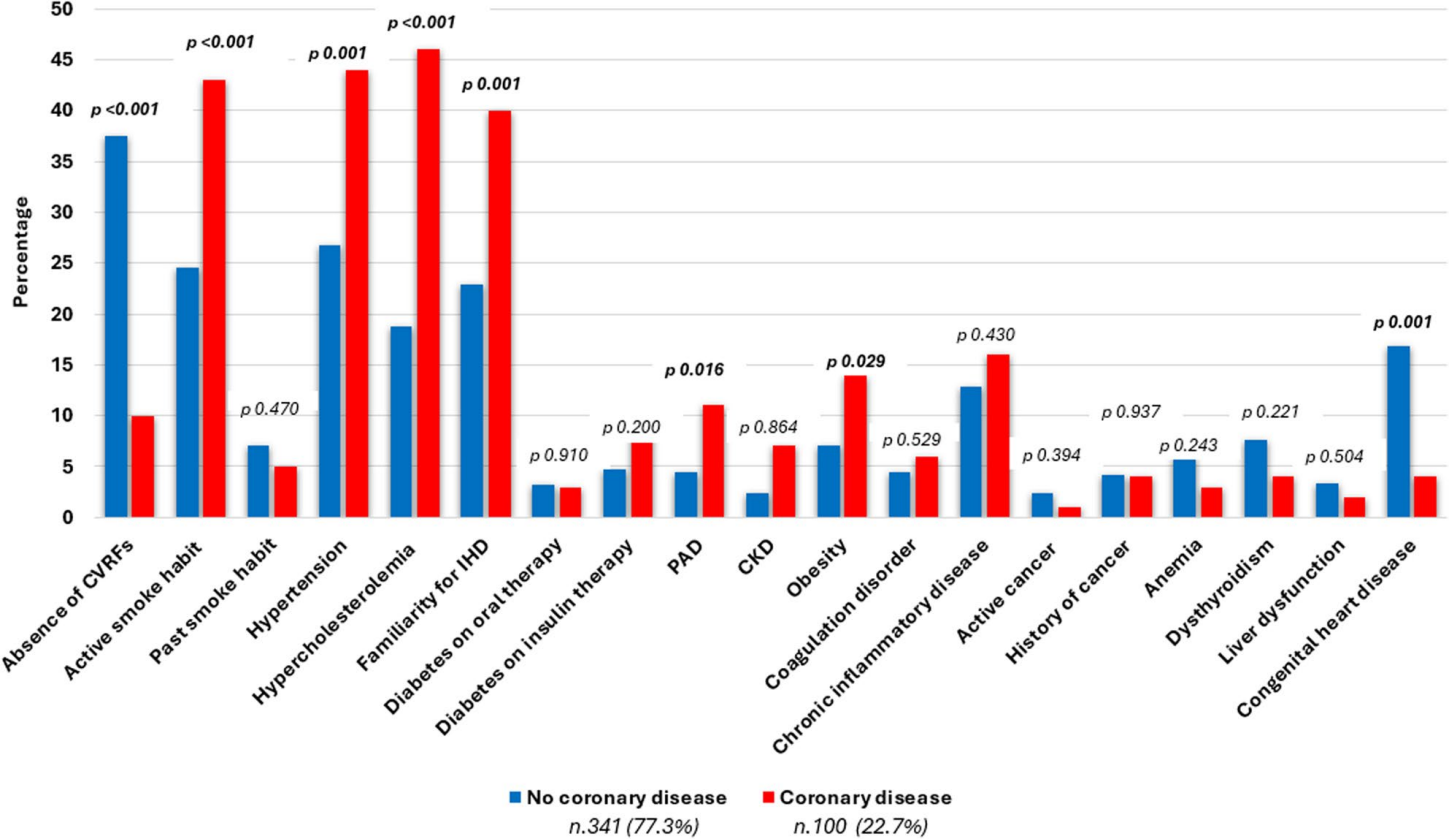
Distribution of traditional and non-traditional risk factors in people < 40-year-old with or without coronary artery disease. CVRFs: cardiovascular risk factors; IHD: ischemic heart disease; PAD: peripheral artery disease; CKD: chronic kidney disease

Table 2 and Fig. 3 summarize CVRFs distribution among the population. The most prevalent CVRFs were hypertension (30.6%), active smoking (28.8%), a family history of ischemic heart disease (26.8%), and hypercholesterolemia (24.9%). The prevalence of diabetes was comparatively lower, with rates of 5.4% for insulin-treated individuals and 3.2% for those on oral therapy. A higher prevalence of all traditional CVRFs was observed in the CAD cohort compared to the no-CAD cohort, encompassing hypertension (44.0% vs. 26.7%, *p* = 0.001), active smoking (43.0% vs. 24.6%, *p* < 0.001), hypercholesterolemia (46.0% vs. 18.8%, *p* < 0.001), family history of IHD (40.0% vs. 22.9%, *p* = 0.001), and obesity (14.0% vs. 7.0%, *p* = 0.029). The prevalence of diabetes was similarly low between the two cohorts (3.0% vs. 3.2%, *p* = 0.910 in oral therapy and 8.0% vs. 4.7%, *p* = 0.200 in insulin therapy). Among individuals with CAD, 10.0% had no traditional CVRFs.

Peripheral artery disease (PAD) was more prevalent in the CAD group vs. no-CAD group (11.1% vs. 4.5%, *p* = 0.016), while chronic kidney disease (7.1% vs. 6.6%, *p* = 0.864), chronic obstructive pulmonary disease (2.0% vs. 2.4%, *p* = 0.818), active cancer (1.0% vs. 2.4%, *p* = 0.394), past cancer history (4.0% vs. 4.2%, *p* = 0.937), coagulation disorder (6.0% vs. 4.5%, *p* = 0.529), chronic inflammatory disease (16.0% vs. 12.9% *p* = 0.430) and recreational drug (4.0% vs. 5.6%, *p* = 0.53) were similarly represented in the two groups (Table 2 and Fig. 3).

The presence of congenital heart disease (4.0% in CAD group vs. 16.8% in no-CAD group, *p* = 0.001) was associated with only diagnostic procedures due to the absence of significant coronary artery disease (Table 2 and Fig. 3).

**Fig. 4:**
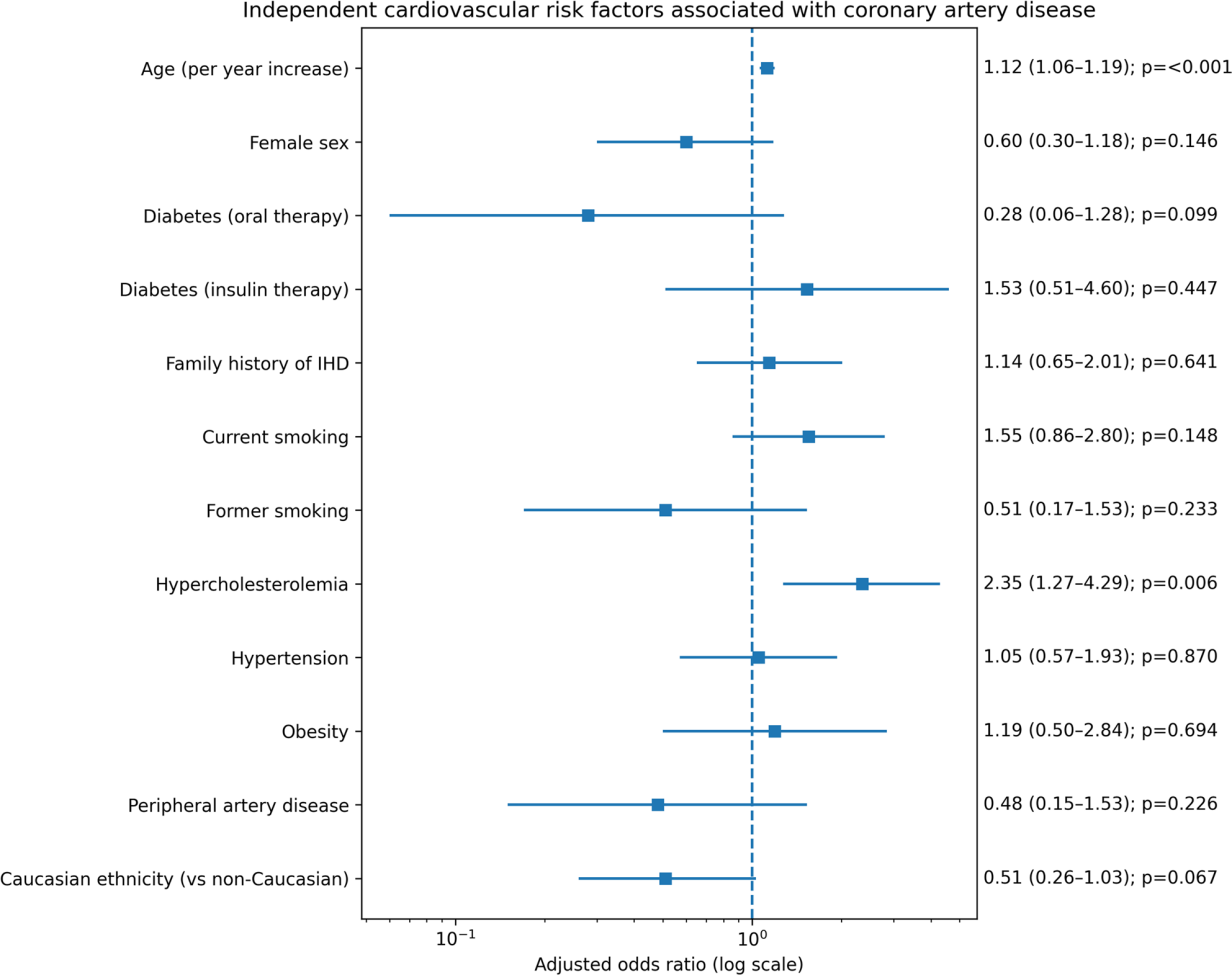
Forest plot of the multivariable logistic regression analysis evaluating independent cardiovascular risk factors and ethnicity associated with coronary artery disease

A multivariable logistic regression analysis was performed to identify independent cardiovascular risk factors associated with the presence of coronary artery disease, including age, sex, ethnicity, and traditional risk factors (Fig. 4). Increasing age was independently associated with CAD (OR 1.12 per year, 95% CI 1.06–1.19, *p* < 0.001). Hypercholesterolemia also emerged as an independent predictor of CAD (OR 2.35, 95% CI 1.27–4.29, *p* = 0.006).

Caucasian ethnicity showed a trend toward a lower likelihood of CAD compared with non-Caucasian ethnicity; however, this association did not reach statistical significance after multivariable adjustment (OR 0.51, 95% CI 0.26–1.03, *p* = 0.067). Sex, diabetes stratified by treatment modality, smoking status, hypertension, obesity, peripheral artery disease, and family history of ischemic heart disease were not independently associated with CAD in the adjusted model.

### Sensitivity analysis

In a sensitivity analysis restricted to the first invasive coronary angiography per patient (*n* = 419), the multivariable logistic regression analysis yielded results consistent with the main procedure-based analysis. Increasing age (OR 1.11 per year; *p* < 0.001) and hypercholesterolemia (OR 2.39; *p* = 0.007) remained independently associated with coronary artery disease. Ethnicity was not independently associated with coronary artery disease after adjustment (OR 0.52; *p* = 0.093).

### Clinical presentation and left ventricular function

**Fig. 5:**
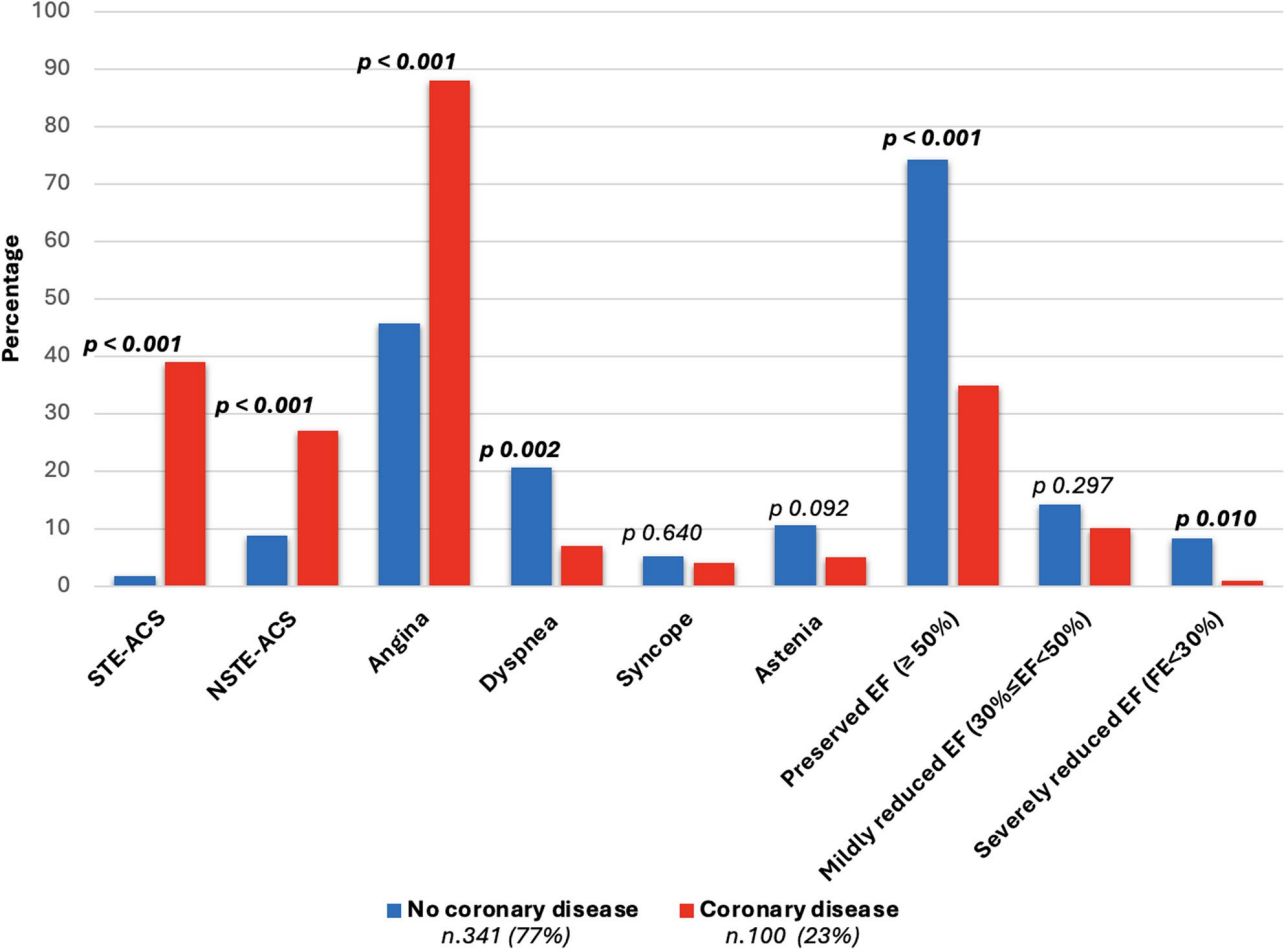
Clinical presentation and left ventricular ejection fraction at admission. EF: ejection fraction

Angina was predominant in CAD group vs. no-CAD patients (88.0% vs. 45.7%, *p* < 0.001), while dyspnea was less prevalent (7.0% vs. 20.6% in CAD vs. no-CAD group, *p* = 0.002) (Table 3 and Fig. 5). 102 (23.1%) patients underwent ICA due to acute coronary syndrome. Among them, 57 (12.9%) presented with non-ST elevation Acute Coronary Syndromes (NSTE-ACS) and 45 (10.2%) were ST elevation Acute Coronary Syndromes (STE-ACS). Among patients presenting with STE-ACS, obstructive CAD was found in 86.7%, compared with 47.4% among those presenting with NSTE-ACS.

**Table 3:** Symptoms and clinical presentation on admission

Clinical presentation and Symptoms	All procedures (441)	No coronary disease (341; 77.3%)	Coronary disease (100; 22.7%)	*p*-value
Clinical presentation: STE-ACS	45 (10.2%)	6 (1.8%)	39 (39.0%)	**< 0.001**
Clinical presentation: NSTE-ACS	57 (12.9%)	30 (8.8%)	27 (27.0%)	**< 0.001**
Dyspnea	77 (17.4%)	70 (20.6%)	7 (7.0%)	**0.002**
Angina	245 (55.6%)	157 (46.0%)	88 (88.0%)	**< 0.001**
Syncope	22 (5.0%)	18 (5.2%)	4 (4.0%)	0.640
Asthenia	41 (9.3%)	36 (10.6%)	5 (5.0%)	0.092
Cardiac arrest	21 (4.9%)	14 (4.2%)	7 (7.0%)	0.259

*Abbreviations: STE-ACS* ST-Elevation Acute Coronary Syndrome, *NSTE-ACS* Non-ST-Elevation Acute Coronary Syndrome

Bold values indicate statistical significance (*p* < 0.05)

Patients with either preserved left ventricular ejection fraction (EF ≥ 50%) (34.9% vs. 74.2%, *p* < 0.001) or severely reduced EF (< 30%) (1.0% in CAD group vs. 8.3% in no-CAD group, *p* = 0.010), were more likely to have non-significant coronary disease whilst no significant differences were detected regarding mildly reduced EF (30%≤ EF < 50%) (10.1% in CAD group vs. 14.2% in no-CAD group, *p* = 0.297). These results are reported in Table 2 and Fig. 5.

**Fig. 6:**
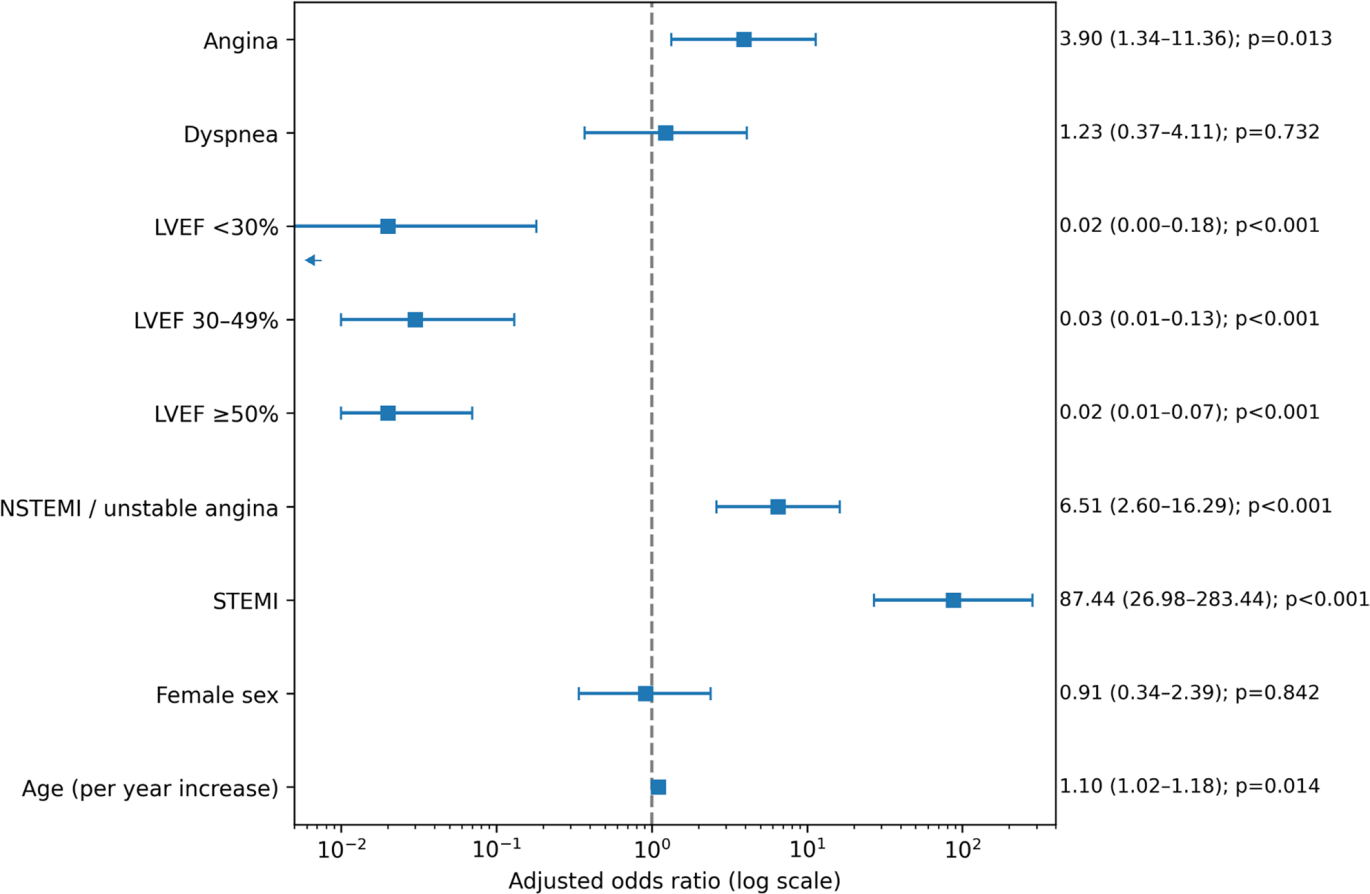
Forest plot of the multivariable logistic regression analysis including clinical presentation and echocardiographic variables associated with the presence of coronary artery disease

A separate multivariable logistic regression model including demographic variables, clinical presentation, and echocardiographic findings was performed to evaluate factors associated with the presence of coronary artery disease at angiography (Fig. 6). Increasing age was independently associated with CAD (OR 1.10 per year, 95% CI 1.02–1.18, *p* = 0.014), whereas sex was not significantly associated with the presence of coronary disease after adjustment.

Among clinical presentation variables, both STE-ACS (OR 87.45, 95% CI 26.2–291.8, *p* < 0.001) and NSTE-ACS (OR 6.51, 95% CI 2.6–16.3, *p* < 0.001) were strongly associated with angiographic CAD. Typical angina symptoms were also independently associated with CAD (OR 3.90, 95% CI 1.34–11.3, *p* = 0.013), whereas dyspnea was not independently associated with the presence of coronary disease.

Compared with mildly reduced ejection fraction, both preserved and severely reduced ejection fraction were independently associated with a lower likelihood of CAD.

### Gender differences

**Fig. 7:**
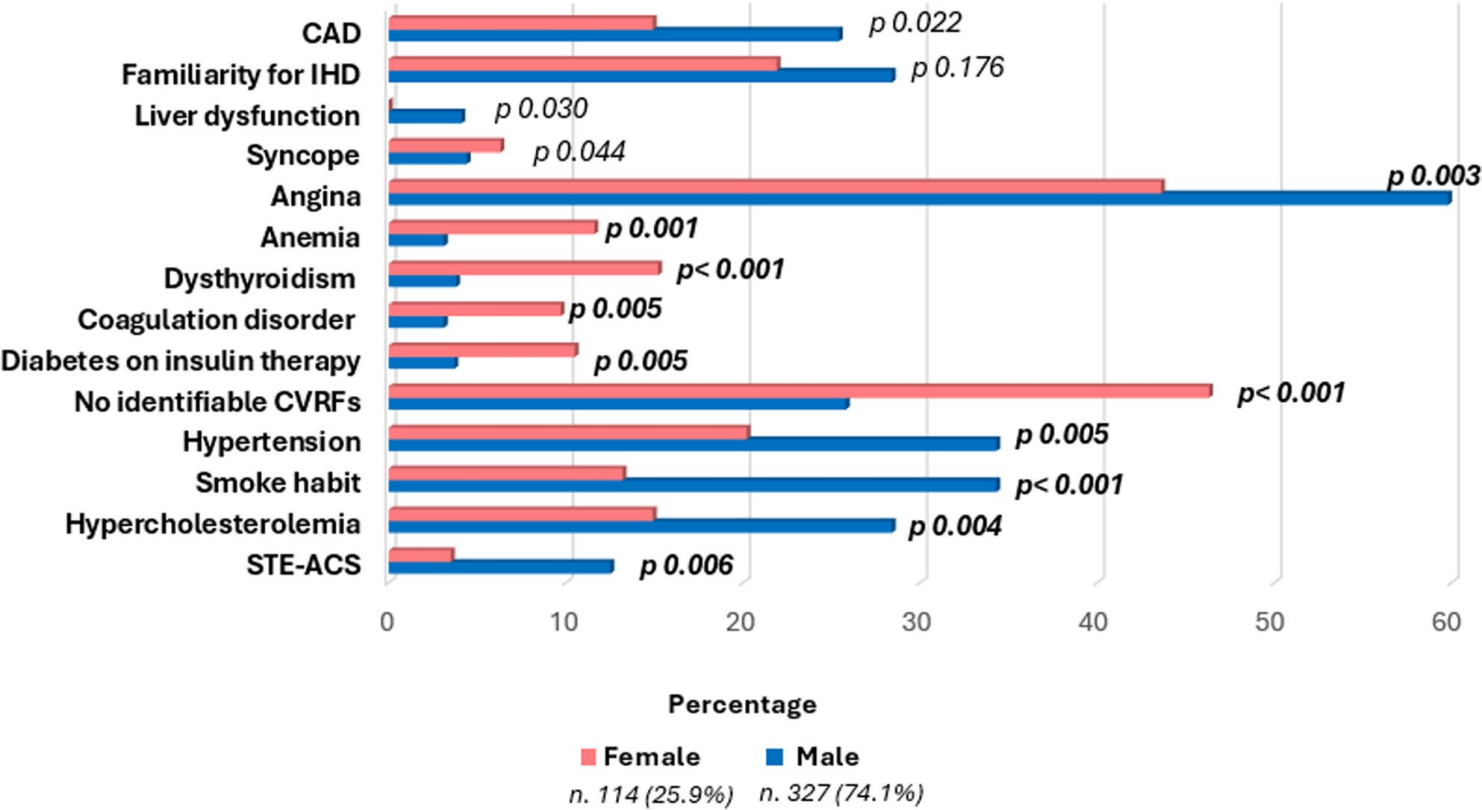
Gender differences in our population

An analysis of gender differences was also performed (Table 4 and Fig. 7). Males had a higher incidence of STE-ACS (12.5% vs. 3.5%, *p* = 0.006) and had higher prevalence of CAD in general (25.4% vs. 14.9%, *p* = 0.022). There were also more frequently hypercholesterolemic, active smoker and hypertensive (28.4% vs. 14.9%, *p* = 0.004; 34.3% vs. 13.2%, *p* < 0.001; 34.3% vs. 20.2%, *p* = 0.005, respectively). Conversely, nearly half of the women had no identifiable CVRFs as compared to men (46.3% vs. 25.8%, *p* < 0.001). In relation to diabetes, diabetes mellitus on insulin therapy was more prevalent among females (10.5% vs. 3.7%, *p* = 0.005), whereas diabetes mellitus on oral therapy was more prevalent in males (4.3% vs. 0.0%, *p* = 0.025). No significant difference was found as regards familiarity for IHD (28.4% vs. 21.9%, *p* = 0.176). The analysis of the comorbidities indicated that females had a greater incidence of coagulation disorders (3.1% vs. 9.7% *p* = 0.005), dysthyroidism (3.8% vs. 15.2%, *p* < 0.001), and anemia (3.1% vs. 11.6%, *p* = 0.001). Conversely, liver dysfunction was observed to be more prevalent among men (4.1% vs. 0.0% *p* = 0.030). Recreational drug use was significantly more frequent in men than in women (6.7% vs. 0.9%, *p* = 0.016). Regarding clinical presentation, angina was more prevalent in males (59.8% in males vs. 43.6% in females, *p* = 0.003), whilst syncope was more prevalent in the female population (4.4% in males vs. 6.3% in females, *p* = 0.044).

**Table 4:** Gender differences in the prevalence of cardiovascular risk factors and other clinical features

Features	Male (327; 74.1%)	Female (114, 25.9%)	p-value
Absence of risk factors	84 (25.8%)	53 (46.3%)	**< 0.001**
Active smoke habit	112 (34.3%)	15 (13.2%)	**< 0.001**
Past smoke habit	19 (5.8%)	10 (8.8%)	0.272
Hypertension	112 (34.3%)	23 (20.2%)	**0.005**
Hypercholesterolemia	93 (28.4%)	17 (14.9%)	**0.004**
Familiarity for IHD	93 (28.4%)	25 (21.9%)	0.176
Diabetes on oral therapy	14 (4.3%)	0 (0.0%)	**0.025**
Diabetes on insulin therapy	12 (3.7%)	12 (10.5%)	**0.005**
Peripheral artery disease	22 (6.6%)	5 (4.5%)	0.414
Chronic kidney disease (eGFR < 60 ml/min/1.73 m^2^)	23 (6.9%)	7 (6.3%)	0.803
Chronic obstructive pulmonary disease	8 (2.5%)	2 (1.8%)	0.659
Obesity	31 (9.5%)	7 (6.1%)	0.274
Coagulation disorder	10 (3.1%)	11 (9.7%)	**0.005**
Chronic inflammatory disease	40 (12.1%)	21 (18.0%)	0.118
Active cancer	5 (1.5%)	4 (3.5%)	0.200
History of cancer	11 (3.4%)	7 (6.3%)	0.193
Use of recreational drug	22 (6.7%)	1 (0.9%)	**0.016**
Heart failure	22 (6.6%)	8 (6.7%)	0.982
Preserved EF (≥ 50%)	212 (64.7%)	75 (65.4%)	0.888
Mildly reduced EF (30 ≤ EF < 50%)	36 (11.0%)	22 (19.6%)	**0.023**
Severely reduced EF (EF < 30%)	22 (6.6%)	8 (6.6%)	0.982
Congenital heart disease	43 (13.1%)	18 (15.9%)	0.451
Anemia	10 (3.1%)	13 (11.6%)	**0.001**
Dysthyroidism	12 (3.8%)	17 (15.2%)	**< 0.001**
Liver dysfunction	13 (4.1%)	0 (0.0%)	**0.030**
Angina	196 (59.8%)	50 (43.6%)	**0.003**
Dyspnea	51 (15.7%)	25 (22.3%)	0.114
Asthenia	30 (9.1%)	11 (9.7%)	0.847
Syncope	14 (4.4%)	7 (6.3%)	**0.044**
CAD	83 (25.4%)	17 (14.9%)	**0.022**
STE-ACS	41 (12.5%)	4 (3.5%)	**0.006**
NSTE-ACS	44 (13.5%)	13 (11.4%)	0.574
One vessel disease	68 (20.8%)	14 (12.4%)	0.064
Two vessels disease	25 (7.6%)	4 (3.9%)	0.192
Three vessels disease	12 (3.8%)	2 (1.9%)	0.358

*Abbreviations: IHD* Ischemic Heart Disease, *eGFR* Estimated Glomerular Filtration Rate, *EF* Ejection Fraction, *CAD* Coronary Artery Disease, *STE-ACS* ST-Elevation Acute Coronary Syndrome, *NSTE-ACS* Non-ST-Elevation Acute Coronary Syndrome

Bold values indicate statistical significance (*p* < 0.05)

### Ethnic differences

**Fig. 8:**
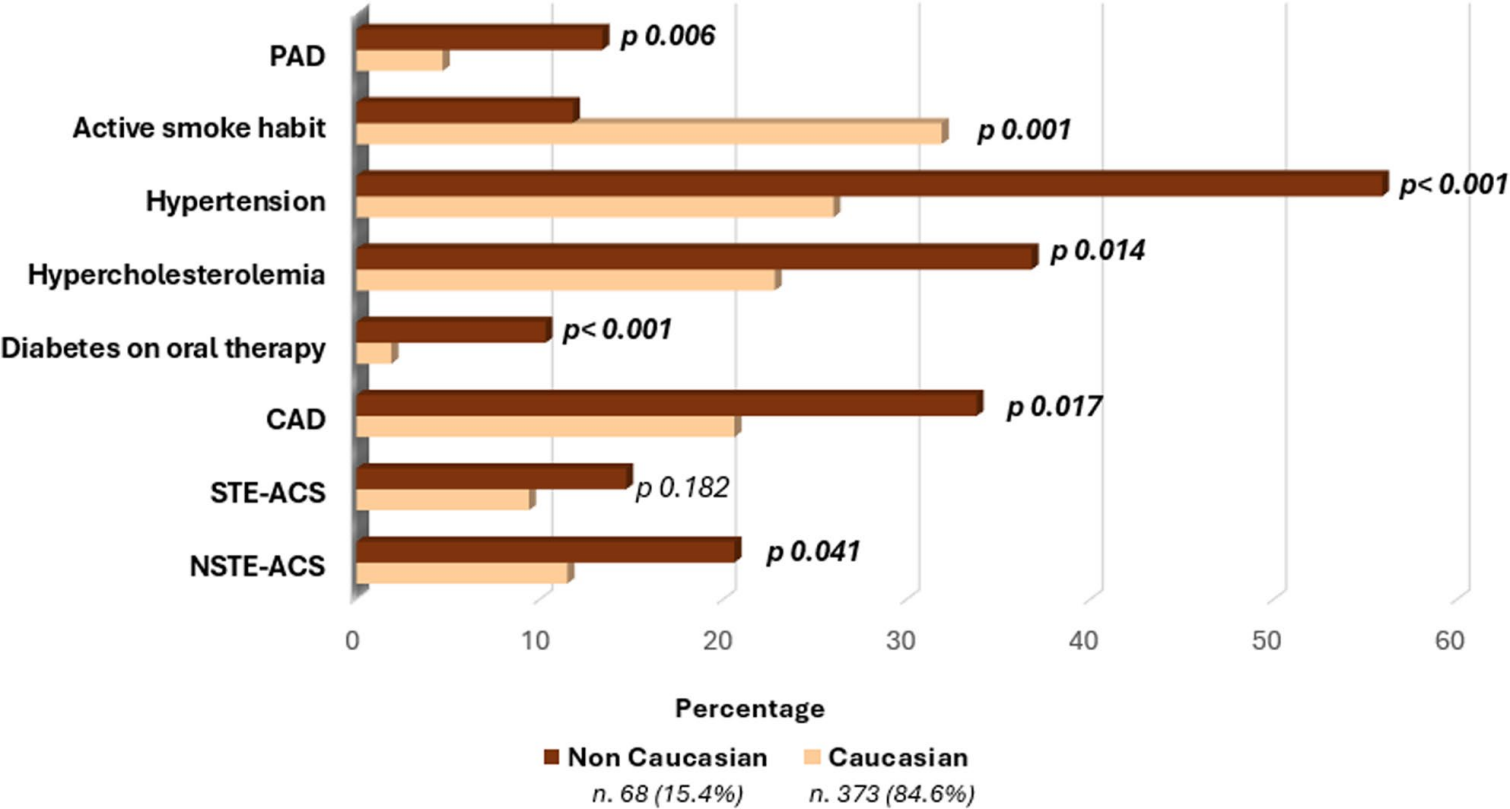
Ethnic differences in our population. CAD: coronary artery disease

Non-Caucasian patients were more frequently affected by diabetes on oral therapy (10.3% vs. 1.9%, *p* < 0.001), hypercholesterolemia (22.8% vs. 36.8%, *p* = 0.014), and hypertension (55.9% vs. 26.0%, *p* < 0.001); on the contrary, active smoking was more represented in Caucasian patients (31.9% vs. 11.8%, *p* = 0.001). No significant differences were observed regarding familiarity for IHD (26.8% vs. 26.5%, *p* = 0.954) and obesity (10.3% vs. 8.3%, *p* = 0.592) between different ethnic groups. Lastly, PAD was more prevalent in non-Caucasian individuals (9.3% vs. 5.1%, *p* = 0.006). These results are summarized in Table 5 and Fig. 8. As per clinical presentation, NSTE-ACS was significantly more frequent in non-Caucasian individuals (20.6% vs. 11.5% *p* = 0.041), whereas STE-ACS were also more prevalent in non-Caucasian individuals, but the difference did not reach statistical significance (14.7% vs. 9.4%, *p* = 0.18). Consequently, CAD was more frequent in non-Caucasian people (33.8% vs. 20.6%, *p* = 0.017) (Table 5 and Fig. 8). Three-vessel CAD was more prevalent in the non-Caucasian population compared to the Caucasian population (7.8% vs. 2.5%, *p* = 0.03). On the contrary, no significant differences were observed in the incidence of one-vessel and two-vessel disease between the groups (Supplementary Fig. 2).

**Table 5:** Ethnic differences in the prevalence of cardiovascular risk factors (CVRFs) and other clinical features

Features	Caucasian (373; 84.6%)	Non Caucasian (68; 15.4%)	*p*-value
Absence of risk factors	119 (32.0%)	15 (21.5%)	0.072
Active smoke habit	119 (31.9%)	8 (11.8%)	**0.001**
Past smoke habit	24 (6.4%)	5 (7.4%)	0.779
Hypertension	97 (26.0%)	38 (55.9%)	**< 0.001**
Hypercholesterolemia	85 (22.8%)	25 (36.8%)	**0.014**
Familiarity for IHD	100 (26.8%)	18 (26.5%)	0.954
Diabetes on oral therapy	7 (1.9%)	7 (10.3%)	**< 0.001**
Diabetes on insulin therapy	20 (5.4%)	4 (5.9%)	0.862
Peripheral artery disease	18 (4.7%)	9 (13.4%)	**0.006**
Chronic kidney disease (eGFR < 60 ml/min/1.73 m^2^)	22 (5.8%)	8 (11.9%)	0.066
Chronic obstructive pulmonary disease	10 (2.8%)	0 (0.0%)	0.169
Obesity	31 (8.3%)	7 (10.3%)	0.592
Coagulation disorder	20 (5.4%)	1 (1.5%)	0.167
Chronic inflammatory disease	53 (14.2%)	7 (10.4%)	0.437
Active cancer	8 (2.2%)	1 (1.5%)	0.721
History of cancer	16 (4.4%)	2 (3.0%)	0.606
Use of recreational drug	19 (5.1%)	4 (5.9%)	0.788
Heart failure	22 (5.9%)	7 (10.8%)	0.144
Preserved EF (≥ 50%)	250 (66.9%)	37 (53.8%)	**0.043**
Mildly reduced EF (30 ≤ EF < 50%)	58 (15.5%)	5 (7.7%)	0.154
Severely reduced EF (EF < 30%)	22 (5.9%)	7 (10.8%)	0.144
Congenital heart disease	48 (13.0%)	12 (18.2%)	0.265
Dysthyroidism	29 (7.7%)	1 (1.5%)	0.062
Anemia	22 (5.8%)	2 (3.0%)	0.349
Angina	208 (55.8%)	38 (55.2%)	0.941
Dyspnea	61 (16.3%)	16 (23.9%)	0.131
Asthenia	30 (8.0%)	11 (16.4%)	**0.028**
Syncope	15 (4.1%)	6 (9.0%)	0.092
CAD	77 (20.6%)	23 (33.8%)	**0.017**
STE-ACS	35 (9.4%)	10 (14.7%)	0.182
NSTE-ACS	43 (11.5%)	14 (20.6%)	**0.041**
One vessel disease	68 (18.1%)	15 (22.2%)	0.444
Two vessels disease	22 (6.0%)	7 (10.9%)	0.144
Three vessel disease	9 (2.5%)	5 (7.8%)	**0.032**

*Abbreviations: IHD* Ischemic Heart Disease, *eGFR* Estimated Glomerular Filtration Rate, *EF* Ejection Fraction, *CAD* Coronary Artery Disease, *STE-ACS* ST-Elevation Acute Coronary Syndrome, *NSTE-ACS* Non-ST-Elevation Acute Coronary Syndrome

Bold values indicate statistical significance (*p* < 0.05)

## Discussion

The present study provides a detailed characterization of clinical presentation and cardiovascular risk factors in very young patients (≤ 40 years) referred for invasive coronary angiography due to suspected coronary artery disease. Traditional cardiovascular risk factors were common in this population, with the exception of diabetes, and 10% of patients with angiographic CAD had no traditional risk factors. Among non-traditional comorbidities, only peripheral artery disease was more frequent in patients with CAD. We also observed clinically relevant differences by sex and ethnicity, with non-Caucasian patients showing a higher burden of several cardiometabolic risk factors.

Data on very young patients undergoing invasive coronary angiography for suspected CAD remain limited. Despite being a single-center study, our cohort represents one of the largest reported cohorts of patients aged ≤ 40 years undergoing ICA. A relevant finding was the high proportion of diagnostic-only procedures, reflecting the low prevalence of obstructive CAD in this age group, consistent with previous reports [10, 16]. This finding suggests that those with normal coronary anatomy may not necessarily be experiencing ACS or chronic coronary syndrome (CCS), but they may have similar symptoms due to other conditions. Conversely, it is worth noting that we included patients since 2010, a time in which non-invasive anatomical tests, such as coronary computed tomography (CT) angiography, were not yet widely adopted. Notably, we observed a progressive reduction in the annual number of invasive procedures over time, possibly reflecting the increasing adoption of non-invasive diagnostic strategies, such as coronary CT angiography. However, other factors, including changes in guideline recommendations, referral patterns, and local diagnostic pathways, may also have contributed to this trend. In this context, dyspnea showed poor association with angiographic CAD in our population. According to the 2024 ESC guidelines on CCS, individuals under 40 years of age with dyspnea, factoring in up to five CVRFs (the maximum for the Risk Factor-weighted Clinical Likelihood (RF-CL) score), are categorized as low or very low risk for CAD. Consequently, it is plausible that a subset of the patients enrolled in the study might have benefited from non-invasive testing rather than immediate invasive angiography.

One of the primary goals of our study was to provide an overview of the prevalence of CVRFs in a tertiary high-volume center. Consistent with several previous studies [6–17], traditional risk factors — including hypertension, active smoking, hypercholesterolemia, family history of coronary artery disease, and obesity— were more prevalent among patients with angiographic CAD. When compared with other single-center cohorts, differences in the burden of cardiovascular risk factors emerge across geographic regions.

In a Polish single-center study involving young patients undergoing coronary angiography [5], the prevalence of most traditional risk factors, particularly dyslipidemia, smoking, and hypertension, was substantially higher than in our cohort. These differences may reflect population-specific characteristics as well as temporal trends, as our study enrolled patients in a more recent era during which a gradual decline in cardiovascular risk factor prevalence has been reported [18]. Additionally, a larger portion of our study population underwent coronary angiography for indications other than ACS (which represented the main indication for coronary angiography in the Polish study), including CCS, heart failure, myocarditis and congenital disease. Despite these differences, our study further confirmed that diabetes is the least prevalent risk factor among younger individuals, a prevalence that is well-documented to be mostly age-related [19, 20].

Similarly, data from the Tawam Hospital cohort [21] which included slightly older patients, showed comparable rates of hypertension and dyslipidemia but a markedly higher prevalence of diabetes and obesity. This finding is likely related to both age differences and regional epidemiological patterns. Notably, diabetes remained the least prevalent risk factor in our cohort, supporting the concept that its impact on coronary artery disease in very young individuals is strongly age-dependent. This highlights the importance of country-specific registries to guide efforts in addressing modifiable risk factors. In our study, data about obesity and diabetes are relatively favorable compared to other countries, potentially reflecting the benefits of the Mediterranean diet [22]. On the other hand, substantial efforts remain necessary to reduce the rates of modifiable risk factors like dyslipidemia and arterial hypertension. The difficulty in achieving effective blood pressure control has led to 2024 ESC guidelines with stricter control measures and the introduction of the new entity of “elevated blood pressure” [23].

In multivariable analysis, increasing age and hypercholesterolemia remained independently associated with angiographic CAD within the constraints of the current model. However, these findings should be interpreted with caution. The relatively limited number of CAD events in this very young cohort restricts the events-per-variable ratio and may reduce the statistical power to detect independent associations for other traditional cardiovascular risk factors.

Moreover, clustering of risk factors such as male sex, smoking, dyslipidemia, and hypertension is common in young patients, and this interdependence may attenuate the apparent independent contribution of individual variables in adjusted models. Importantly, lack of statistical significance does not imply lack of biological relevance. The well-established role of smoking, hypertension, and family history in atherosclerosis remains unquestioned, and their contribution to the overall cardiovascular risk profile in this population should not be underestimated.

Given the growing recognition that factors beyond traditional cardiovascular risk factors may contribute to coronary artery disease in younger populations, we also evaluated the role of non-traditional comorbidities. Among these, only peripheral artery disease was more prevalent in patients with angiographic CAD, while chronic kidney disease, chronic obstructive pulmonary disease, malignancy, coagulation disorders, and chronic inflammatory diseases were not associated with CAD.

The association between peripheral artery disease and coronary artery disease in our cohort supports the concept of an early and systemic atherosclerotic burden, even at a young age. Although PAD is not currently included in tools estimating the pre-test probability of CAD, its relevance is increasingly recognized, as reflected in the emphasis on peripheral and carotid artery assessment in the 2024 ESC guidelines on chronic coronary syndromes [24]. These findings are consistent with previous longitudinal studies demonstrating the prognostic significance of early vascular disease in young individuals, including the Muscatine Study [25] and the Cardiovascular Risk in Young Finns Study [26].

Sex-related differences emerged clearly in our cohort. In this context, it is worth noting that males were significantly more prevalent than their counterparts, constituting 74% as compared to 26%, particularly in the context of ACS, similarly with the findings of previous studies [27]. Males exhibited a higher likelihood of presenting with common CVRFs, while nearly half of the women displayed no identifiable risk factors. Females were more prone to conditions such as dysthyroidism and anemia, which may confound symptoms like dyspnea.

These findings suggest that traditional risk stratification based on cardiovascular risk factors may be less effective in young women, potentially contributing to differences in clinical presentation and referral patterns. Our results were consistent with previous studies on ACS [28], that reported a higher prevalence in men. In a study by Revaiah et al. [1], gender difference was so pronounced that 96.2% of ACS men aging less or 40 years were men. In a large Swiss prospective cohort study, Schoenenberger et al. [27] enrolled almost 29.000 patients with ACS, finding a young (< 35 years) female gender prevalence of 14.9%, significantly lower than that observed in older age groups (*p* < 0.001). Together, these observations highlight the need for sex-specific awareness in the evaluation of young patients presenting with suspected coronary syndromes.

To the best of our knowledge, this is the first European study exploring ethnic differences in cardiovascular risk factors, clinical presentation, and angiographic findings among very young patients undergoing invasive coronary angiography. Although the sample size was insufficient to draw definitive conclusions, we hypothesize that ethnicity may act as a surrogate for differences in cardiometabolic risk profiles, access to care, and social or environmental determinants. However, the non-Caucasian group likely represented a heterogeneous population with different geographic and ethnic backgrounds, and therefore the observed associations should be interpreted with caution. In this context, ethnicity should be considered mainly as a surrogate marker for cardiometabolic, social, and environmental determinants rather than a causal factor per se. Notably, we observed a significantly higher prevalence of NSTE-ACS cases among non-Caucasian individuals, as well as a higher prevalence of CAD and specifically three-vessel disease. This aligns with data from American studies [29] showing that African American males have the highest incidence rates of MI, succeeded by African American females, Caucasian males, and Caucasian females across all ages, including young adults. However, ethnicity was not independently associated with coronary artery disease after multivariable adjustment, suggesting that the observed differences may be mediated by the unequal distribution of cardiovascular risk factors and clinical presentation.

The last important result of our study involves the clinical presentation. The majority of patients with coronary disease were admitted for angina (88.0%). Dyspnea was less common (7.0%), followed by asthenia (5.0%) and syncope (4.0%). The prominence of angina in both acute and chronic settings has been well documented in other studies; however, its elevated prevalence among younger patients deserves particular attention. Additionally, those presenting with dyspnea were less likely to have coronary disease. These findings were further supported by multivariable analysis, in which acute coronary syndromes and typical angina emerged as the strongest independent predictors of angiographic coronary artery disease. The magnitude of these associations should be interpreted in the context of an invasive cohort, in which patients presenting with acute coronary syndromes are more likely to undergo coronary angiography and to have obstructive disease identified. Therefore, these effect estimates likely reflect selection characteristics of an ICA-based population rather than a predictive effect applicable to the broader community.

Nevertheless, this observation remains consistent with contemporary European guidelines, which attribute increasing diagnostic weight to angina symptoms within the risk-factor-modified clinical likelihood model [24]. In this sense, our findings reinforce the clinical relevance of symptom characterization in young patients referred for invasive evaluation.

The finding that both preserved and severely reduced ejection fraction were associated with a lower likelihood of angiographic CAD may appear counterintuitive. However, in very young patients, severe left ventricular dysfunction is often related to alternative diagnoses such as myocarditis, dilated cardiomyopathy, congenital heart disease, or other non-ischemic conditions. Conversely, patients with preserved ejection fraction may be referred for invasive angiography because of symptoms or suspected ischemia, but ultimately show normal or non-obstructive coronary arteries. These factors may explain the higher proportion of non-significant coronary findings in these subgroups.

This study has several limitations. Its retrospective and single-center design may limit generalizability. Clinical outcomes were not systematically collected and therefore could not be evaluated. In addition, the absence of systematic functional coronary assessment may have led to an underestimation of non-obstructive coronary disease, including conditions such as coronary vasospasm or microvascular dysfunction. Moreover, systematic intracoronary imaging was not available, and some etiological misclassification cannot be completely excluded. Although data completeness for major cardiovascular risk factors was high and procedures with incomplete clinical or angiographic data were excluded, some degree of residual misclassification cannot be entirely excluded due to the retrospective design and reliance on medical record documentation rather than standardized prospective assessment. In addition, as some patients underwent more than one invasive coronary angiography before the age of 40, a sensitivity analysis restricted to the first procedure per patient was performed, yielding results consistent with the main analysis and supporting the robustness of our findings. However, some degree of residual within-patient correlation cannot be completely excluded. Moreover, ethnicity was recorded as a dichotomous variable (Caucasian vs. non-Caucasian), and the non-Caucasian group likely encompassed a heterogeneous mix of populations with diverse genetic, cultural, and socioeconomic backgrounds. As such, ethnicity should not be interpreted as a causal determinant of coronary disease but rather as a surrogate marker potentially reflecting differences in cardiometabolic burden, healthcare access, lifestyle factors, and broader social determinants of health. This simplified categorization may limit the external validity and mechanistic interpretation of these findings.

In addition, regarding the multivariable models, the relatively limited number of CAD events (*n* = 100 procedures) may have constrained statistical power, particularly when multiple covariates were included. The events-per-variable ratio and potential clustering among traditional risk factors (e.g., smoking, dyslipidemia, and male sex) may have attenuated the apparent independent contribution of individual variables. These factors should be considered when interpreting the adjusted estimates. Furthermore, it should be acknowledged that the absence of statistical significance should not be equated with absence of biological effect. Lastly, we acknowledge that cardiovascular risk factor profiles may differ between patients presenting with ACS and those with CCS, and that the lack of a direct subgroup comparison (ACS vs. stable CAD) is an important limitation of the present study: while we present clinical-presentation–adjusted models, a formal, separate multivariable comparison was not performed and remains an important subject for future analyses.

Taken together, our findings should be interpreted within the evolving landscape of premature coronary artery disease, where traditional risk factors remain central but are increasingly accompanied by emerging contributors—such as psychosocial stress, environmental exposures, dietary patterns characterized by ultra-processed foods, elevated lipoprotein(a), and recreational drug use [30–34], underscoring the need for a more comprehensive and forward-looking preventive approach in young individuals.

## Conclusions

Coronary artery disease represents a clinically relevant condition also among very young individuals, in whom data remain limited. In this large single-center cohort of patients aged ≤ 40 years undergoing invasive coronary angiography, traditional cardiovascular risk factors were highly prevalent and strongly associated with the presence of angiographic coronary artery disease. In multivariable analyses, increasing age and hypercholesterolemia emerged as the only independent predictors of CAD, underscoring their central role in early atherosclerotic disease.

Notably, a substantial proportion of young patients with CAD had no traditional cardiovascular risk factors, highlighting the need for alternative and complementary risk assessment strategies in this population. Although non-Caucasian ethnicity was associated with a higher burden of cardiovascular risk factors, more severe angiographic disease, and a higher prevalence of acute coronary syndromes at univariable analysis, ethnicity was not independently associated with CAD after multivariable adjustment, suggesting that these differences are largely mediated by the unequal distribution of cardiometabolic risk factors and clinical presentation.

Finally, clinical presentation played a pivotal role in identifying patients with angiographic CAD, with acute coronary syndromes and typical angina emerging as the strongest independent predictors, while dyspnea was poorly associated with obstructive disease. These findings support a more selective and individualized diagnostic approach in very young patients, integrating age, lipid profile, and symptom characteristics to optimize risk stratification and guide the use of invasive coronary angiography.

## Supplementary Information

Supplementary Material 1: Supplementary Figure 1 Temporal trend in the number of Caucasian and non-Caucasian patients aged ≤40 years undergoing invasive coronary angiography from 2010 to 2024.

Supplementary Material 2: Supplementary figure 2 Ethnic Differences in angiography findings.

## Data Availability

The data and materials underlying this article will be shared on reasonable request to the corresponding author.

## Abbreviations

ACS: Acute Coronary Syndrome
ARIC: Atherosclerosis Risk in Communities
CABG: Coronary Artery Bypass Graft Surgery
CAD: Coronary Artery Disease
Cath-lab: Catheterization Laboratory
CCS: Chronic Coronary Syndrome
CVD: Cardiovascular Disease
CVRFs: Cardiovascular Risk Factors
DALYs: Disability-Adjusted Life Years
EF: Ejection Fraction
ICA: Invasive Coronary Angiography
IHD: Ischemic Heart Disease
MI: Myocardial Infarction
NSTE-ACS: Non-ST-Elevation Acute Coronary Syndrome
PAD: Peripheral Artery Disease
PCI: Percutaneous Coronary Intervention
RF-CL: Risk Factor-weighted Clinical Likelihood
SCD: Sudden Cardiac Death
SPSS: Statistical Package for the Social Sciences
STE-ACS: ST-Elevation Acute Coronary Syndrome
WHO: World Health Organization

## References

[1] Revaiah PC, Vemuri KS, Vijayvergiya R, Bahl A, Gupta A, Bootla D, et al. Epidemiological and clinical profile, management and outcomes of young patients (≤ 40 years) with acute coronary syndrome: A single tertiary care center study. Indian Heart J. 2021;73:295–300. 34154745 10.1016/j.ihj.2021.01.015 PMC8322929

[2] Gaziano TA, Bitton A, Anand S, Abrahams-Gessel S, Murphy A. Growing epidemic of coronary heart disease in low- and middle-income countries. Curr Probl Cardiol. 2010;35:72–115. 20109979 10.1016/j.cpcardiol.2009.10.002 PMC2864143

[3] Bęćkowski M, Gierlotka M, Gąsior M, Poloński L, Zdrojewski T, Dąbrowski R, et al. Risk factors predisposing to acute coronary syndromes in young women ≤ 45 years of age. Int J Cardiol. 2018;264:165–9. 29655953 10.1016/j.ijcard.2018.03.135

[4] Liang MT, Pang Y, Gao LL, Han LJ, Yao HC. Clinical risk factors and outcomes of young patients with acute ST segment elevation myocardial infarction: a retrospective study. BMC Cardiovasc Disord. 2023;23:353. 37460997 10.1186/s12872-023-03392-8 PMC10353156

[5] Maroszyńska-Dmoch EM, Wożakowska-Kapłon B. Clinical and angiographic characteristics of coronary artery disease in young adults: a single centre study. Kardiol Pol. 2016;74:314–21. 26365941 10.5603/KP.a2015.0178

[6] Alkhawam H, Sogomonian R, El-Hunjul M, Kabach M, Syed U, Vyas N, et al. Risk factors for coronary artery disease and acute coronary syndrome in patients ≤ 40 years old. Future Cardiol. 2016;12:545–52. 27492147 10.2217/fca-2016-0011

[7] Yusuf S, Hawken S, Ounpuu S, Dans T, Avezum A, Lanas F, et al. Effect of potentially modifiable risk factors associated with myocardial infarction in 52 countries (the INTERHEART study): case-control study. Lancet. 2004;364(9438):937-52. 10.1016/S0140-6736(04)17018-9 15364185

[8] Enas EA, Dhawan J, Petkar S. Coronary artery disease in Asian Indians: lessons learnt and the role of lipoprotein(a). Indian Heart J. 1997;49:25–34. 9130422

[9] Kannel WB, Abbott RD. Incidence and prognosis of unrecognized myocardial infarction. An update on the Framingham study. N Engl J Med. 1984;311:1144–7. 6482932 10.1056/NEJM198411013111802

[10] Fournier JA, Sánchez A, Quero J, Fernández-Cortacero JA, González-Barrero A. Myocardial infarction in men aged 40 years or less: a prospective clinical-angiographic study. Clin Cardiol. 1996;19:631–6. 8864336 10.1002/clc.4960190809

[11] Garoufalis S, Kouvaras G, Vitsias G, Perdikouris K, Markatou P, Hatzisavas J, et al. Comparison of angiographic findings, risk factors, and long term follow-up between young and old patients with a history of myocardial infarction. Int J Cardiol. 1998;67:75–80. 9880203 10.1016/s0167-5273(98)00194-6

[12] Waldmann V, Karam N, Bougouin W, Sharifzadehgan A, Dumas F, Narayanan K, et al. Burden of Coronary Artery Disease as a Cause of Sudden Cardiac Arrest in the Young. J Am Coll Cardiol. 2019;73:2118–20. 31023437 10.1016/j.jacc.2019.01.064

[13] Zheng ZJ, Croft JB, Giles WH, Mensah GA. Sudden cardiac death in the United States, 1989 to 1998. Circulation. 2001;104:2158–63. 11684624 10.1161/hc4301.098254

[14] Arora S, Stouffer GA, Kucharska-Newton AM, Qamar A, Vaduganathan M, Pandey A, et al. Twenty Year Trends and Sex Differences in Young Adults Hospitalized With Acute Myocardial Infarction. Circulation. 2019;139:1047–56. 30586725 10.1161/CIRCULATIONAHA.118.037137 PMC6380926

[15] Agrawal A, Lamichhane P, Eghbali M, Xavier R, Cook DE, Elsherbiny RM, et al. Risk factors, lab parameters, angiographic characteristics and outcomes of coronary artery disease in young South Asian patients: a systematic review. J Int Med Res. 2023;51:3000605231187806. 37555333 10.1177/03000605231187806 PMC10413899

[16] Zimmerman FH, Cameron A, Fisher LD, Ng G. Myocardial infarction in young adults: angiographic characterization, risk factors and prognosis (Coronary Artery Surgery Study Registry). J Am Coll Cardiol. 1995;26:654–61. 7642855 10.1016/0735-1097(95)00254-2

[17] Konishi H, Miyauchi K, Kasai T, Tsuboi S, Ogita M, Naito R, et al. Long-term prognosis and clinical characteristics of young adults (≤ 40 years old) who underwent percutaneous coronary intervention. J Cardiol. 2014;64:171–4. 24495504 10.1016/j.jjcc.2013.12.005

[18] World health statistics 2023: monitoring health for the SDGs, Sustainable Development Goals. Geneva: World Health Organization; 2023. Licence: CC BY-NC-SA 3.0 IGO.

[19] Disoteo O, Grimaldi F, Papini E, Attanasio R, Tonutti L, Pellegrini MA, et al. State-of-the-Art Review on Diabetes Care in Italy. Ann Glob Health. 2015;81:803–13. 27108147 10.1016/j.aogh.2015.12.013

[20] Gnavi R, Migliardi A, Maggini M, Costa G. Prevalence of and secular trends in diagnosed diabetes in Italy: 1980–2013. Nutr Metab Cardiovasc Dis. 2018;28:219–25. 29337018 10.1016/j.numecd.2017.12.004

[21] Jamil S, Jamil G, Mesameh H, Qureshi A, AlKaabi J, Sharma C, et al. Risk factor comparison in young patients presenting with acute coronary syndrome with atherosclerotic coronary artery disease vs. angiographically normal coronaries. Int J Med Sci. 2021;18:3526–32. 34522179 10.7150/ijms.60869 PMC8436094

[22] Visseren FLJ, Mach F, Smulders YM, Carballo D, Koskinas KC, Bäck M, et al. 2021 ESC Guidelines on cardiovascular disease prevention in clinical practice. Eur Heart J. 2021;42:3227–337. 34458905 10.1093/eurheartj/ehab484

[23] McEvoy JW, McCarthy CP, Bruno RM, Brouwers S, Canavan MD, Ceconi C, et al. 2024 ESC Guidelines for the management of elevated blood pressure and hypertension. Eur Heart J. 2024;45:3912–4018. 39210715 10.1093/eurheartj/ehae178

[24] Vrints C, Andreotti F, Koskinas KC, Rossello X, Adamo M, Ainslie J, et al. 2024 ESC Guidelines for the management of chronic coronary syndromes. Eur Heart J. 2024;45:3415–537. 39210710 10.1093/eurheartj/ehae177

[25] Burns TL, Letuchy EM, Paulos R, Witt J. Childhood predictors of the metabolic syndrome in middle-aged adults: the Muscatine study. J Pediatr. 2009;155:Se517–26. 10.1016/j.jpeds.2009.04.044 PMC2739835 19732563

[26] Koskinen JS, Kytö V, Juonala M, Viikari JSA, Nevalainen J, Kähönen M, et al. Childhood Dyslipidemia and Carotid Atherosclerotic Plaque in Adulthood: The Cardiovascular Risk in Young Finns Study. J Am Heart Assoc. 2023;12:e027586. 36927037 10.1161/JAHA.122.027586 PMC10122878

[27] Schoenenberger AW, Radovanovic D, Stauffer JC, Windecker S, Urban P, Niedermaier G, et al. Acute coronary syndromes in young patients: presentation, treatment and outcome. Int J Cardiol. 2011;148:300–4. 19942306 10.1016/j.ijcard.2009.11.009

[28] Cheema FM, Cheema HM, Akram Z. Identification of risk factors of acute coronary syndrome in young patients between 18–40 years of age at a teaching hospital. Pak J Med Sci. 2020;36:821–4. 32494281 10.12669/pjms.36.4.2302 PMC7260892

[29] Benjamin EJ, Blaha MJ, Chiuve SE, Cushman M, Das SR, Deo R, et al. Heart Disease and Stroke Statistics-2017 Update: A Report From the American Heart Association. Circulation. 2017;135:e146–603. 28122885 10.1161/CIR.0000000000000485 PMC5408160

[30] Padmanathan P, Roberts E. Rare but relevant: Cannabis use and myocardial infarction. Addiction. 2025;120:2580–4. 40590409 10.1111/add.70128 PMC12586771

[31] Stătescu C, Anghel L, Benchea LC, Tudurachi BS, Leonte A, Zăvoi A, et al. A Systematic Review on the Risk Modulators of Myocardial Infarction in the Young—Implications of Lipoprotein (a). Int J Mol Sci. 2023;24:5927. 36983001 10.3390/ijms24065927 PMC10051886

[32] Gauci S, Lotfaliany M, Machado P, Hodge A, Gamage E, Levy RB, et al. Exposure to ultra-processed food and risk of cardiovascular mortality: a prospective cohort study. Eur J Prev Cardiol. 2025;32:1564–72. 40561116 10.1093/eurjpc/zwaf378

[33] Sagheer U, Al-Kindi S, Abohashem S, Phillips CT, Rana JS, Bhatnagar A, et al. Environmental Pollution and Cardiovascular Disease. JACC: Adv. 2024;3:100805. 38939391 10.1016/j.jacadv.2023.100805 PMC11198409

[34] Zaheen M, Pender P, Dang QM, Sinha E, Chong JJH, Chow CK, et al. Myocardial Infarction in the Young: Aetiology, Emerging Risk Factors, and the Role of Novel Biomarkers. J Cardiovasc Dev Dis. 2025;12:148. 40278206 10.3390/jcdd12040148 PMC12028068

