# Supplementary figures and images for: Risk factors and clinical presentation of coronary artery disease in young patients: a decade-long single-center experience
Source: BMC Cardiovasc Disord. 2026 Apr 9;26:435. doi: 10.1186/s12872-026-05729-5 (PMC13214127; doi:10.1186/s12872-026-05729-5)

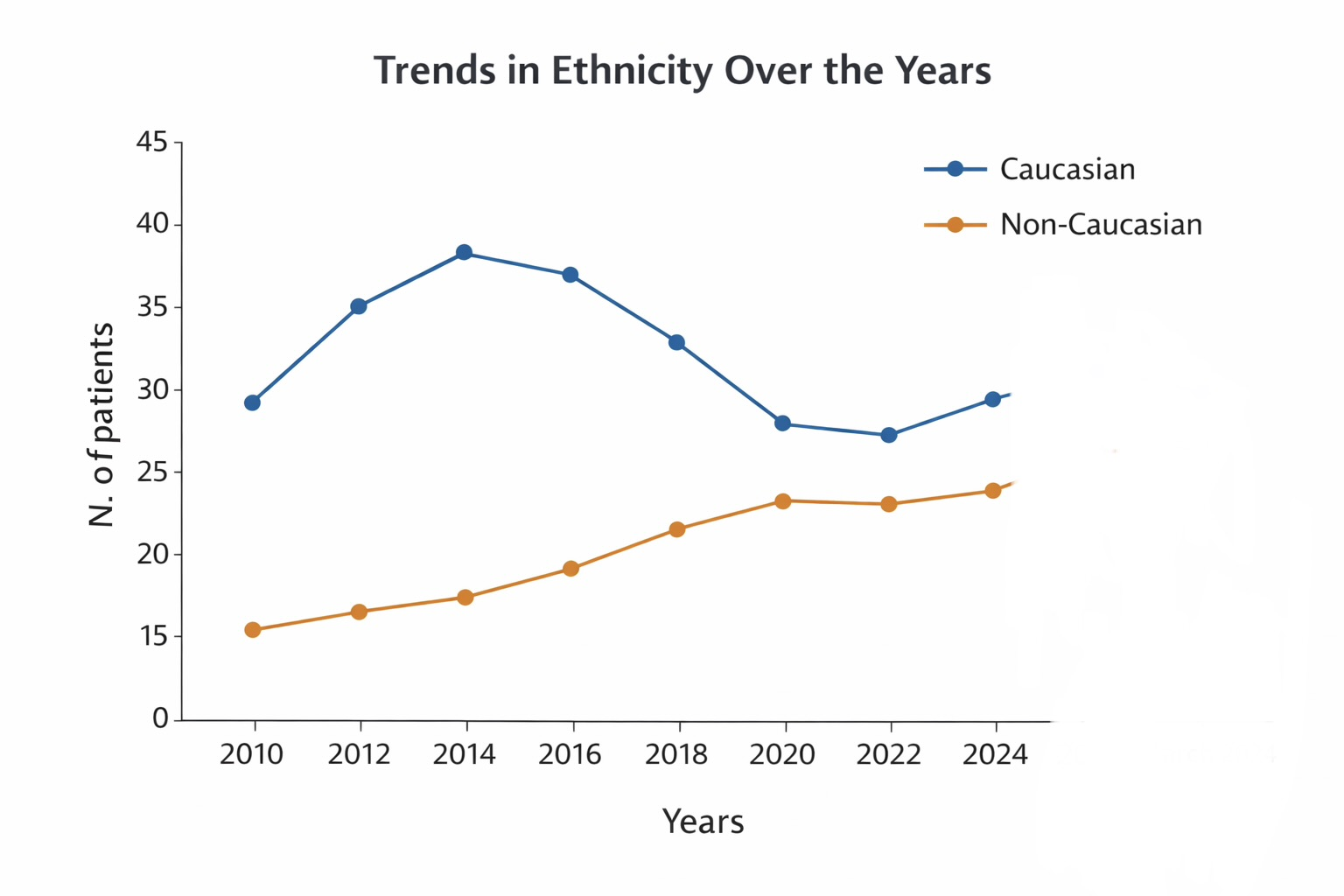

Supplement: Supplementary file 1 — Supplementary Material 1: Supplementary Figure 1 Temporal trend in the number of Caucasian and non-Caucasian patients aged ≤40 years undergoing invasive coronary angiography from 2010 to 2024. [file 12872_2026_5729_MOESM1_ESM.tiff]

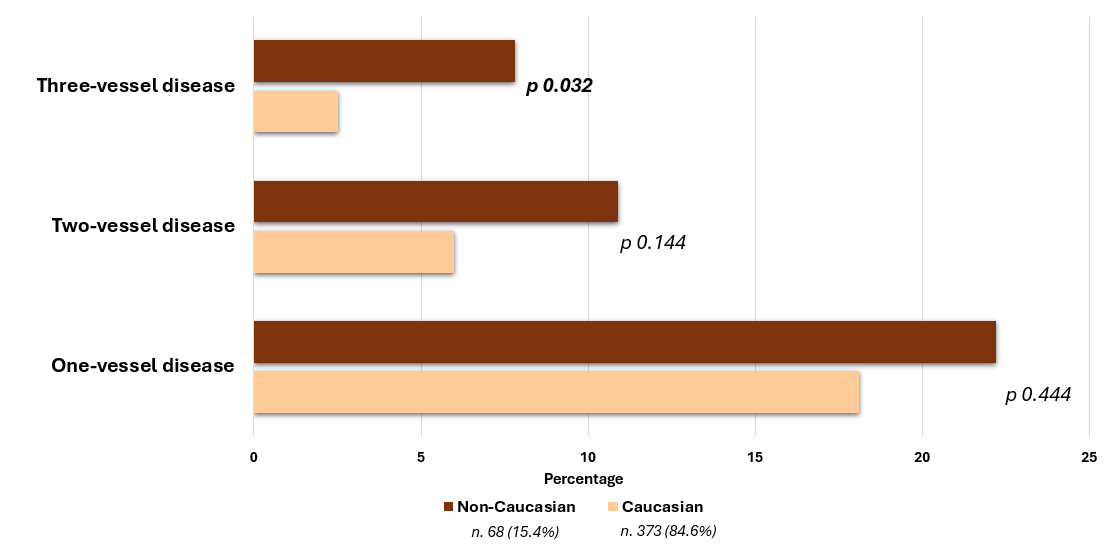

Supplement: Supplementary file 2 — Supplementary Material 2: Supplementary figure 2 Ethnic Differences in angiography findings. [file 12872_2026_5729_MOESM2_ESM.tiff]
